# Supplementary material for: Towards symmetry driven and nature inspired UV filter design
Source: Nat Commun. 2019 Oct 18;10:4748. doi: 10.1038/s41467-019-12719-z (PMC6802189; doi:10.1038/s41467-019-12719-z)
Supplement: Supplementary file 1 — Supplementary Information [file 41467_2019_12719_MOESM1_ESM.pdf]

Supplementary Information

Towards Symmetry Driven and Nature Inspired UV Filter Design

Horbury *et al.*

Supplementary Figures

The transient absorption spectrum/spectra (TAS) for DES in alkyl benzoate (AB, C12–15), ethanol (EtOH) and cyclohexane excited at their UVA λ_{max} 335, 336 and 325 nm respectively, are shown in Supplementary Figures 1-3. The resulting rate constants are shown in Supplementary Table 1.

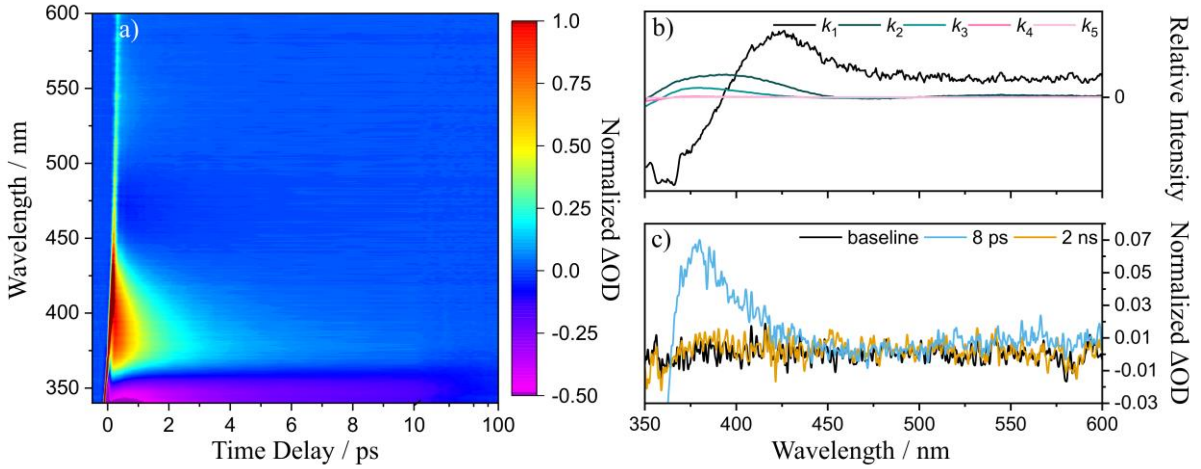

Supplementary Figure 1. **Ultrafast spectroscopy results for DES in AB.** a) Transient absorption spectrum (TAS) of DES in AB photoexcited at 335 nm, shown as a false colourmap, with the intensity scale representing change in normalized optical density (ΔOD). The time-delay is plotted linearly from -0.5 to 10 ps then as a log scale from 10 to 100 ps. b) Evolution associated difference spectra from the sequential global fit of the TAS of DES in AB photoexcited at 335 nm. c) Selected TAS at specific Δ*t* highlighting the absorption at 380 nm (8 ps, blue) and incomplete ground state bleach recovery (2 ns, orange).

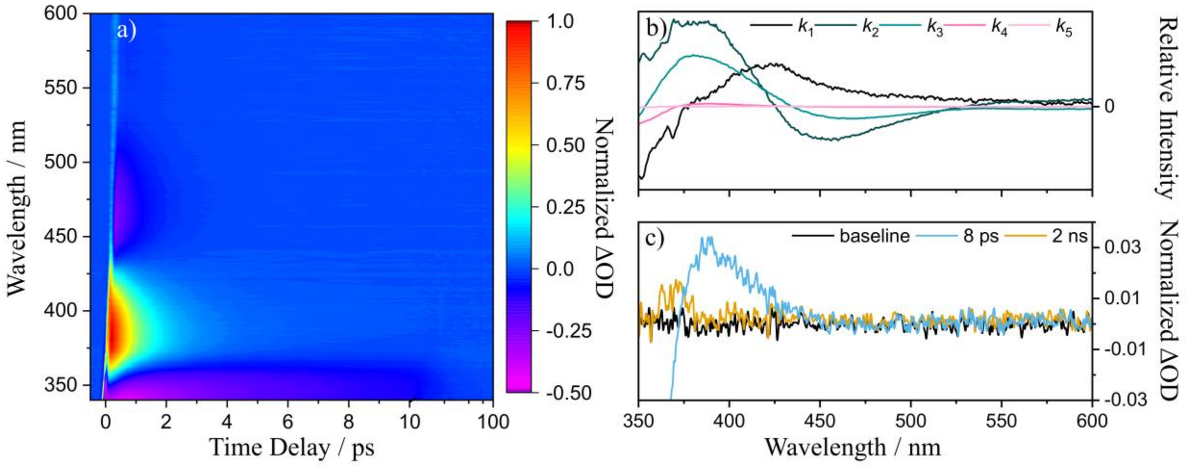

Supplementary Figure 2. **Ultrafast spectroscopy results for DES in ethanol (EtOH).** a) TAS of DES in EtOH photoexcited at 336 nm, shown as a false colourmap, with the intensity scale representing change in normalized optical density (ΔOD). The time-delay is plotted linearly from -0.5 to 10 ps then as a log scale from 10 to 100 ps. b) Evolution associated difference spectra from the sequential global fit of the TAS of DES in EtOH photoexcited at 336 nm. c) Selected TAS at specific Δ*t* highlighting the absorption at 380 nm (8 ps, blue) and evidence of a long-lived photoproduct (2 ns, orange).

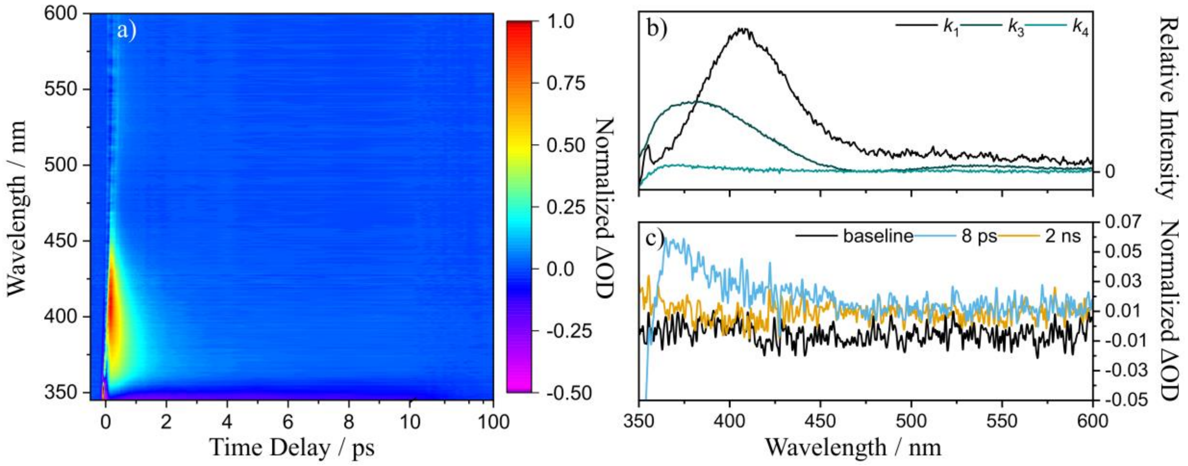

Supplementary Figure 3: **Ultrafast spectroscopy results for DES in cyclohexane.** a) TAS of DES in cyclohexane photoexcited at 325 nm, shown as a false colourmap, with the intensity scale representing change in normalized optical density (ΔOD). The time-delay is plotted linearly from -0.5 to 10 ps then as a log scale from 10 to 100 ps. b) Evolution associated difference spectra from the sequential global fit of the TAS of DES in cyclohexane photoexcited at 325 nm. c) Selected TAS at specific Δ*t* highlighting the absorption at 380 nm (8 ps, blue) and lack of a photoproduct (2 ns, orange).

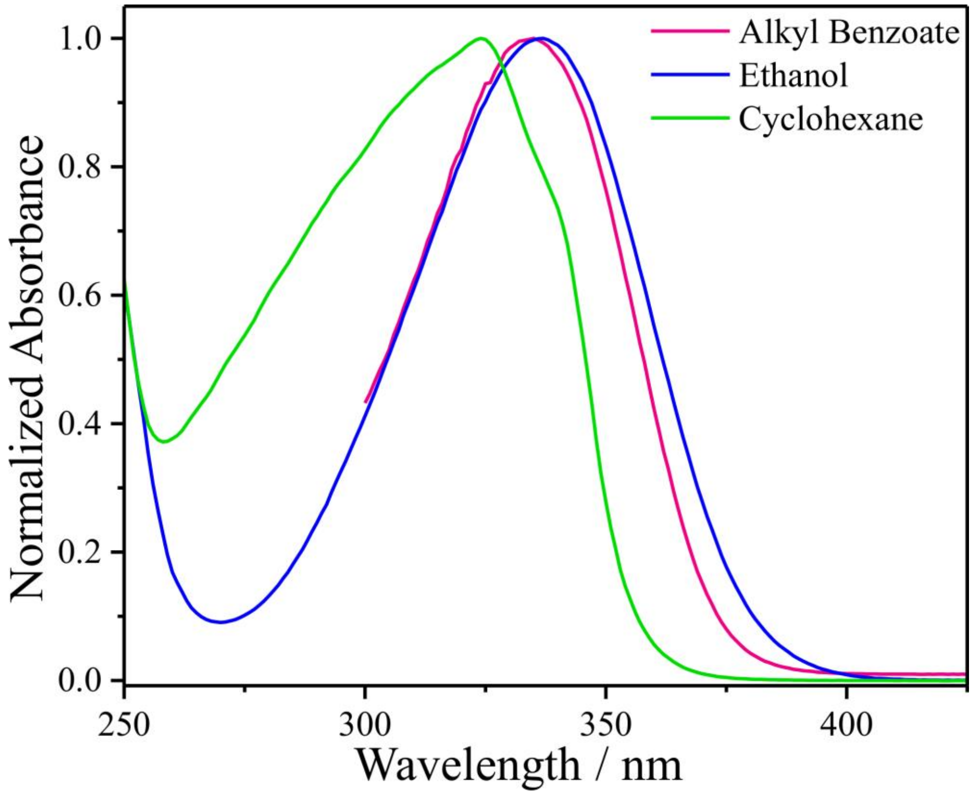

Supplementary Figure 4: **Absorbance region of DES in various solvents.** Ultraviolet/visible spectra of diethyl 2-(4-hydroxy-3,5-dimethoxybenzylidene)malonate (DES**,** ~1 µM) in, C12–15 alkyl benzoate (magenta), ethanol (blue) and cyclohexane (green). We note the cut off in absorption of DES in alkyl benzoate below 300 nm, as the absorption of alkyl benzoate saturates the spectrometer.

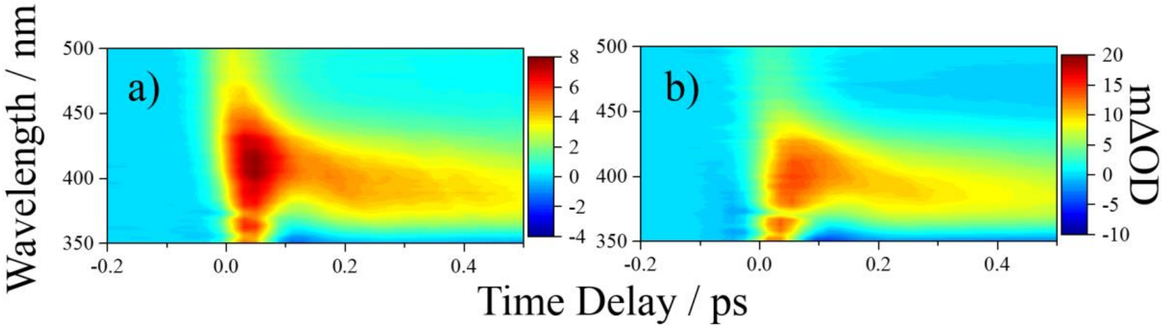

Supplementary Figure 5: **Zoomed-in excited state absorption plots.** False colour map of the transient absorption spectra of DES in a) AB on the surface of VITRO-CORNEUM® (VC) and b) AB, showing the initial blue shifting of the excited state absorption.

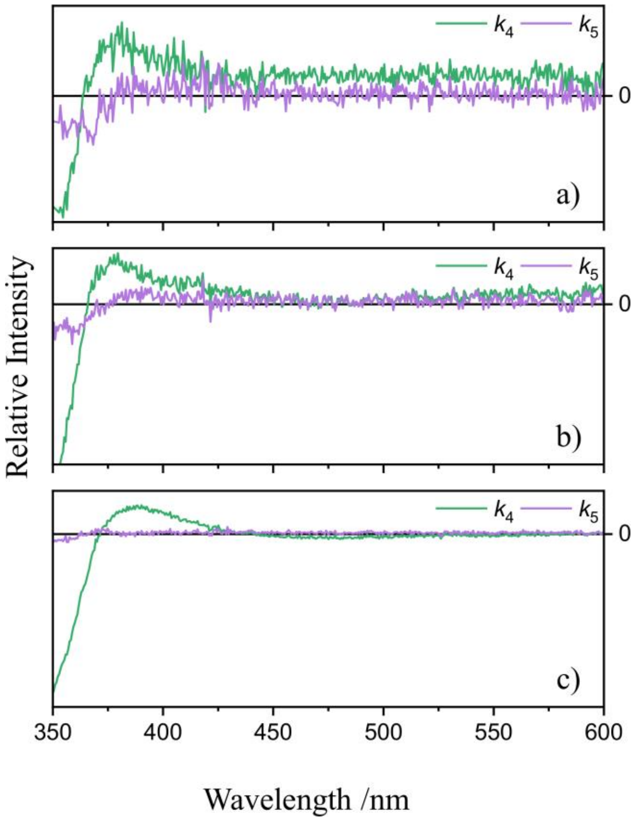

Supplementary Figure 6: **Evolution associated difference spectra (EADS) of k_4 and k_5 only.** Zoomed-in plots of EADS k_4 and k_5 for DES in a) VC/AB, b) AB and c) ethanol.

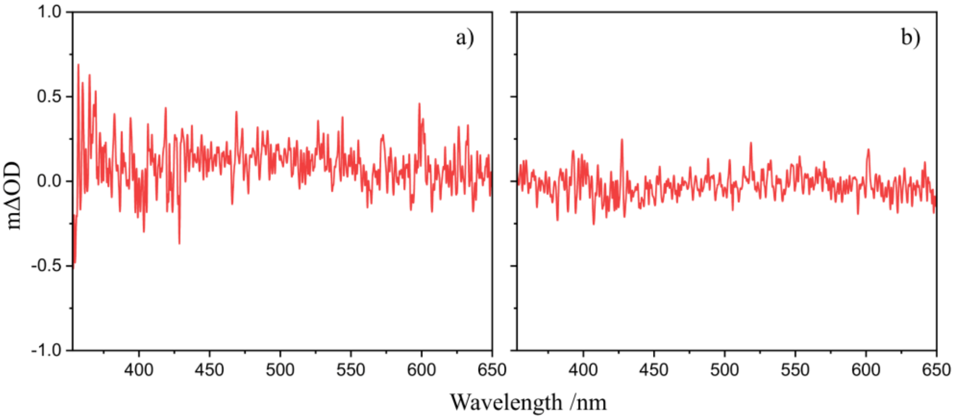

Supplementary Figure 7: Solvent-only responses. TAS of a) VC/AB and b) AB at 1 ps.

To account for the chirp of our probe pulse, a third order polynomial is included within the fitting algorithm. Additionally, the fit is convoluted with an instrument response function (IRF) to account for the temporal resolution of our pulses, whose value is taken from Gaussian fits of the solvent-only time zero response (see Supplementary Figure 8).

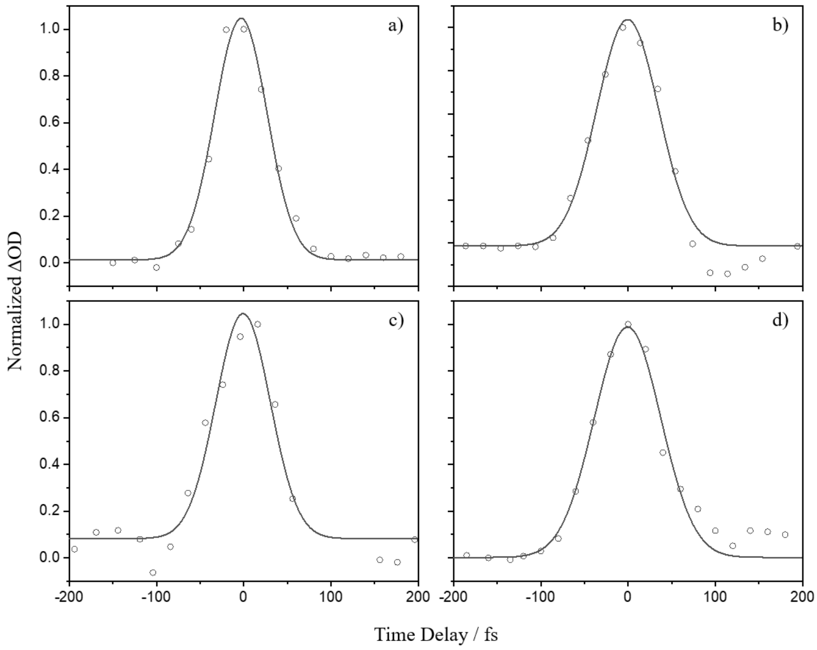

Supplementary Figure 8: **Instrument responses in all solvents.** Selected transients for solvent-only time-zero responses of a) VC/AB (380 nm), b) AB (380 nm), c) ethanol (310 nm) and d) cyclohexane (360 nm). The probe wavelength was chosen which showed the most Gaussian-like response. The solid line represents the fitted to the experimental data with a Gaussian function. The extracted full width half maxima are: a) 80 fs, b) 80 fs, c) 80 fs and d) 90 fs. These values are used as our IRFs in the corresponding global fits.

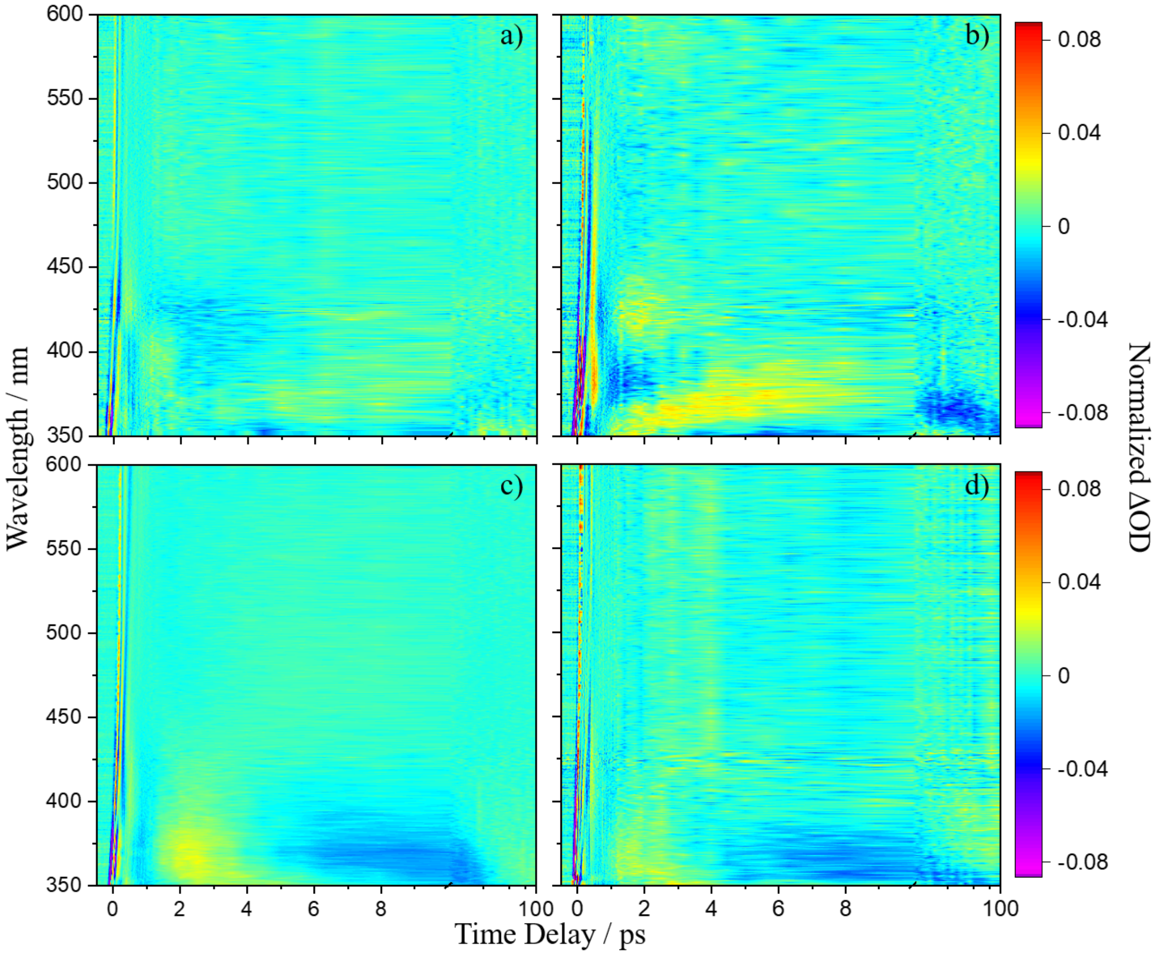

Supplementary Figure 9: **False colour maps of fitting residuals.** Residuals from the global fit of DES in a) VC/AB, b) AB, c) ethanol and d) cyclohexane.

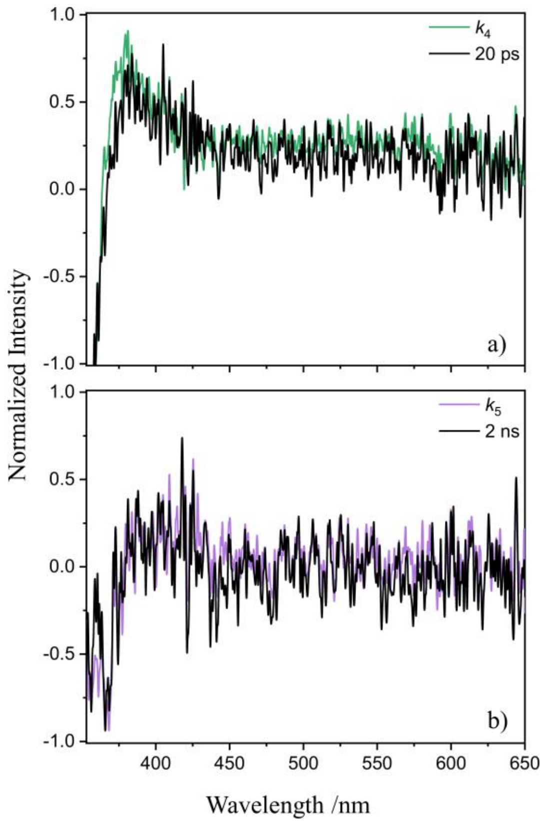

Supplementary Figure 10: **TAS of DES in VC/AB overlaid on EADS.** a) TAS at 20 ps overlaid on the EADS associated with k_4, and b) TAS at 2 ns overlaid with the EADS associated with k_5. Note normalisation is based on the largest amplitude feature.

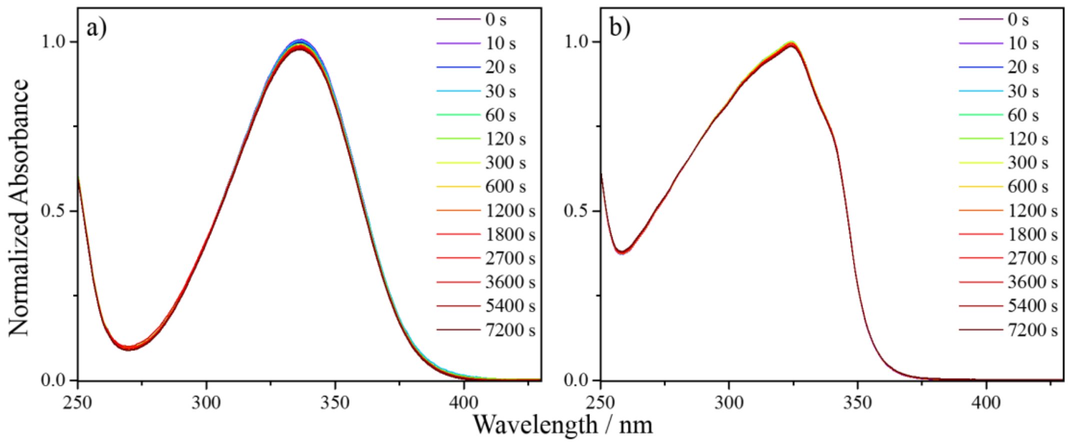

Supplementary Figure 11: Additional solar irradiance studies. UV/visible spectra of DES in a) ethanol and b) cyclohexane at varying durations of irradiation at 336 and 325 nm respectively at solar intensity. The percentage reduction in the UVA λ_{max} was 3.1% and 1.6% respectively.

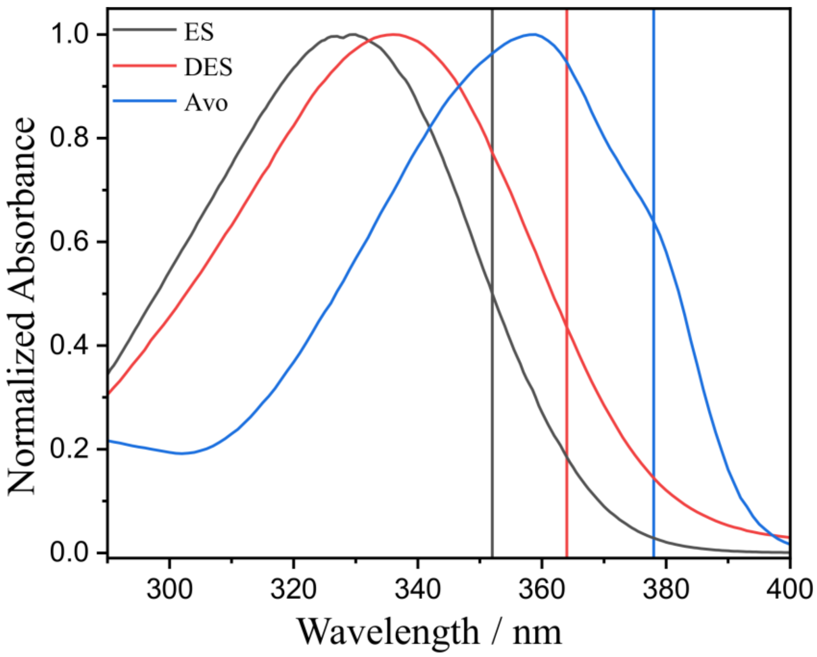

Supplementary Figure 12: Critical wavelengths. UV/visible spectra of DES (red), ES (black) and avobenzone (blue, Avo) in ethanol. The calculated critical wavelengths are DES = 364 nm, ES = 352 nm and Avo = 378 nm, marked by the vertical line in the corresponding colour.

Supplementary Table

Supplementary Table 1: Summary of rate constants. Rate-constants (k_n) resulting from the sequential global fit of the TAS of DES in VC/AB, AB, ethanol and cyclohexane shown in Supplementary Figures 2,3 and 4 respectively. The errors are quoted to 2σ.

	k_1 / s^{-1} (x10^{13})	k_2 / s^{-1} (x10^{12})	k_3 / s^{-1} (x10^{11})	k_4 / s^{-1} (x10^{10})	k_5 / s^{-1} (x10^{8})
VC/AB	0.7 ± 0.2	3.0 ± 0.3	4.24 ± 0.07	1.02 ± 0.06	>> 5
AB	2.5 ± 2.5	2.1 ± 0.2	5.3 ± 0.1	2.6 ± 0.1	>> 5
Ethanol	1.1 ± 0.5	11 ± 5	12.6 ± 0.6	15.5 ± 0.1	>> 5
Cyclohexane	0.7 ± 0.2	n/a	16 ± 1	6.3 ± 0.2	n/a

Supplementary Methods

The critical wavelength for a sunscreen is defined as the wavelength at which the integrated area underneath the spectral absorbance curve reaches 90% of the total area between 290 and 400 nm[1]. To attain the critical wavelengths of DES, ES and avobenzone (Avo), UV/visible spectra of each compound were taken in ethanol using a UV/visible spectrometer (Cary 60, Agilent Technologies). These spectra are shown in Supplementary Fig. 12. The area under each absorption curve between 290 and 400 nm was determined using the cumulative trapezoidal method function in MATLAB (R2017b), which is defined mathematically as follows:

$$\int_{290}^{400} f(\lambda)d\,\lambda \approx \sum_{k=1}^{N} \frac{f(\lambda_{k-1}) + f(\lambda_k)}{2} \Delta x_k \qquad (1)$$

where $\lambda_0 = 290$ nm $< \lambda_1 < \ldots < \lambda_{N-1} < \lambda_N = 400$ nm, and Δx_k is the interval between each wavelength datapoint. The critical wavelength was then assigned to be the value where 90% of the total area resides under the curve. The critical wavelength is marked by a vertical line in Supplementary Fig. 12.

The successful synthesis of DES was confirmed by ^{1}H NMR; the assignments of the peaks are given as follows:

^{1}H NMR (300 MHz, $CDCl_3$): δ = 7.62 (s, 1H, H-1), 6.75 (s, 2H, H-3 and H-7), 4.30 (q, J = 7.23 Hz, 4H, H-16 and H-13), 3.86 (s, 6H, H-9 and H-10), 1.31 (t, J = 7.14 Hz, 6H, H-14 and H-17). ^{13}C NMR (75 MHz, $CDCl_3$): δ = 167.2 (s, C-15), 164.3 (s, C-12), 146.9 (d, C-1), 142.2 (d, C-4 and C-6), 137.3 (s, C-5), 124.0 (s, C-2), 123.8 (s, C-11), 106.64 (d, C-3 and C-7), 61.6 (t, C-16), 61.4 (t, C-13), 56.1 (q, C-9 and C-10), 14.1 (q, C-17), 13.9 (q, C-14).

For the endocrine disruption measurements, cell culture material was from Life Technologies (Cergy-Pontoise, France) except the 96-well Cell star plates, which were from Greiner Labortechnic (Poitiers, France). Luciferin (sodium salt) and geneticin were purchased from Promega (Charbonnières, France). R1881 was from NEN Life Science Products (Paris, France). Estradiol, SR12813, hygromycin and puromycin were purchased from Sigma Aldrich (Saint-Quentin Fallavier, France). Stock solutions were made in dimethyl sulfoxide (DMSO) at 10 mM and dilutions from this stock solution were prepared in a culture medium.

HELN and HELN hERα cells were already described.[2] Briefly, Hela cells were stably transfected with the ERE-βGlobin-Luciferase-SVNeomycin plasmid, with or without the pSG5-hERα-puromycin plasmid leading to the HELN and HELN hERα-cell lines. HG5LN and HG5LN PXR cells were already described.[3] The Hela cells were stably transfected with the $GAL4RE_5$-βGlobin-Luciferase-SVNeomycin plasmid, with or without the pSG5-GAL4(DBD)-hPXR(LBD)-puromycin plasmid leading to the HG5LN and HG5LN-hPXR cell lines.

Cells were cultured at 37°C under humidified 5% CO_2 atmosphere. HG5LN, HG5LN PXR and HELN cells were cultured in red phenol (DMEM)-F12 medium (Thermofisher, Villebon sur Yvette, France) supplemented with 1% penicillin/streptomycin and 5% fetal calf serum (FCS). HELN hERα cells were cultured in DMEM-F12 without red phenol supplemented with penicillin/streptomycin (1%) and dextran-coated charcoal-treated fetal calf serum (DCC-FCS) (5%) (Test medium)

Cells were seeded in 96-well white opaque flat bottom plates at 25,000 cells per well in 150 µL of test medium. DES (four replicates per plate) was added 24h later using automated workstation (Biomek 3000, Beckman Coulter, Villepinte, Paris) and cells were incubated at 37°C for 16h. Then, the medium was removed and 50 µL of test medium containing luciferin at 0.3 mM was added per well. After 5

min, the production of light was measured in living cells using microplate luminometer (MicroBeta, PerkinElmer SAS, Courtaboeuf, France).

Agonistic activities of HELN hERα and HG5LN hPXR cells were tested in the presence of increasing concentrations (10nM-10μM) of DES. Results were expressed as a percentage of maximal luciferase activity. Maximal luciferase activity (100%) was obtained in the presence of 10 nM E2 for ERα, and 3 μM SR12813 for PXR. Antagonistic assays were performed using a concentration of agonist yielding approximately 60-85% of maximal luciferase activity. The antagonistic activity of DES tested at (10 nM – 10 μM) was determined by co-incubation with the agonist E2 at 0.1nM for ERα, and the agonist SR12813 at 100 nM for PXR.

DES was also tested for non-specific modulation of luciferase expression on the HELN and HG5LN cell line, which are devoid of hERα and hPXR. DES showed non-specific induction of luciferase expression at 10 μM.

Supplementary References

1. Diffey BL, Tanner PR, Matts PJ, Nash JF, In vitro assessment of the broad-spectrum ultraviolet protection of sunscreen products. *J. Am. Acad. Dermatol.* **6**, 1024-1035 (2000)
2. Delfosse V*, et al.* Structural and mechanistic insights into bisphenols action provide guidelines for risk assessment and discovery of bisphenol A substitutes. *Proc. Natl. Acad. Sci. USA* **109**, 14930-14935 (2012).
3. Delfosse V*, et al.* Synergistic activation of human pregnane X receptor by binary cocktails of pharmaceutical and environmental compounds. *Nat. Commun.* **6**, 8089 (2015).
